# Rapid Activation of Rac GTPase in Living Cells by Force Is Independent of Src

**DOI:** 10.1371/journal.pone.0007886

**Published:** 2009-11-18

**Authors:** Yeh-Chuin Poh, Sungsoo Na, Farhan Chowdhury, Mingxing Ouyang, Yingxiao Wang, Ning Wang

**Affiliations:** 1 Department of Mechanical Science and Engineering, University of Illinois at Urbana-Champaign, Urbana, Illinois, United States of America; 2 Department of Bioengineering, University of Illinois at Urbana-Champaign, Urbana, Illinois, United States of America; Dresden University of Technology, Germany

## Abstract

It is well known that mechanical forces are crucial in regulating functions of every tissue and organ in a human body. However, it remains unclear how mechanical forces are transduced into biochemical activities and biological responses at the cellular and molecular level. Using the magnetic twisting cytometry technique, we applied local mechanical stresses to living human airway smooth muscle cells with a magnetic bead bound to the cell surface via transmembrane adhesion molecule integrins. The temporal and spatial activation of Rac, a small guanosine triphosphatase, was quantified using a fluorescent resonance energy transfer (FRET) method that measures changes in Rac activity in response to mechanical stresses by quantifying intensity ratios of ECFP (enhanced cyan fluorescent protein as a donor) and YPet (a variant yellow fluorescent protein as an acceptor) of the Rac biosensor. The applied stress induced rapid activation (less than 300 ms) of Rac at the cell periphery. In contrast, platelet derived growth factor (PDGF) induced Rac activation at a much later time (>30 sec). There was no stress-induced Rac activation when a mutant form of the Rac biosensor (RacN17) was transfected or when the magnetic bead was coated with transferrin or with poly-L-lysine. It is known that PDGF-induced Rac activation depends on Src activity. Surprisingly, pre-treatment of the cells with specific Src inhibitor PP1 or knocking-out Src gene had no effects on stress-induced Rac activation. In addition, eliminating lipid rafts through extraction of cholesterol from the plasma membrane did not prevent stress-induced Rac activation, suggesting a raft-independent mechanism in governing the Rac activation upon mechanical stimulation. Further evidence indicates that Rac activation by stress depends on the magnitudes of the applied stress and cytoskeletal integrity. Our results suggest that Rac activation by mechanical forces is rapid, direct and does not depend on Src activation. These findings suggest that signaling pathways of mechanical forces via integrins might be fundamentally different from those of growth factors.

## Introduction

It is now clear that mechanical forces play vital roles in shaping the normal functions of all tissues and organs of human beings [1]. What is not known, however, is by what mechanisms mechanical forces affect tissue and organ functions. Specifically, it is not clear how mechanical forces are converted into biochemical signals inside the living cells; i.e., the mechanism of mechanotransduction. Over the years, several models of mechanotransduction have been proposed such as stretch-activated membrane ion channel opening and local plasma membrane protein unfolding [2]. The main thrust of these models is that mechanotransduction, similar to the soluble factor induced signal transduction, initiates at the cell membrane by inducing local conformational changes or unfolding of membrane-bound proteins at the site of a local force, followed by a cascade of diffusion and translocation processes for downstream signaling. This is consistent with the theory of the classical continuum mechanics of St. Venant's principle that a local force must cause only a local deformation.

As for the soluble factor induced signal transduction, it has been shown recently that platelet derived growth factor (PDGF) activates Src kinase [3], which in turn, leads to activation of Rac [4]. If Rac activation by mechanical forces were similar to that by PDGF, as predicted by the dominant mechanotransduction model, one would expect the stress-induced Rac activation to be dependent also on Src activation. In sharp contrast, if Rac GTPase could be directly activated by mechanical forces at the cell surface, then its activation would not depend on Src activity. In this study, we examined whether Rac can be directly activated by stress independent of Src.

## Materials and Methods

### Cell Culture and Reagents

Human airway smooth muscle (HASM) cells were isolated at autopsy within 8 hrs of death from tracheal muscle of lung transplant donors (approved by the University of Pennsylvania Committee on studies involving human beings) at University of Pennsylvania in Dr. Panettieri's laboratory [5].

#### Ethics Statement

We used de-identified HASM cells supplied by Dr. Panettieri who obtained the tissue through NDRI (National Disease Research Interchange) in a manner that excludes all unique identifying information. There is no consent form sent with the tissue as per NDRI. All our procedures were approved by the Institutional Review Board of University of Illinois at Urbana-Champaign.

A monoclonal antibody that recognizes only the α- and β-isoactin of smooth muscle was used to identify the cells as smooth muscle cells. Cells were cultured at a density of 10,000 cells/cm^2^ with Ham/F12 media, supplemented with 10% fetal bovine serum, 50 µg/ml gentamicin, and 2.5 µg/ml amphotericin B. When cells reached passage two, they were shipped for further culturing and experiments. Cells at passage 3–8 were used for all experiments. These cells still maintain smooth muscle cell morphology and physiological responsiveness to agonists at passage 8. After cells reached confluence in culture dishes, they were serum deprived for 24 h before being trypsinized. Following trypsinization, cells were plated in serum free medium (IT, i.e. Insulin-Transferrin containing medium) overnight in 35-mm dishes for experiments. HASM cells do not enter cell cycle but maintain contractile profile in IT medium. The Src/Yes/Fyn triple-knockout (SYF−/−) mouse embryonic fibroblast (MEF) cells were cultured and maintained in DMEM (Sigma) supplemented with 10% fetal bovine serum (HyClone), 100 U/ml penicillin, 100 µg/ml streptomycin, and 2 mM L-Glutamine at 37°C in 5% CO_2_. The 35-mm dishes (No. 00, VWR) were pre-coated with collagen-1 (0.02 mg/ml) in phosphate-buffered saline (PBS) to facilitate absorption of protein onto the wells. Then the cells were plated in the wells at ∼7,000 cells/well for live cell imaging. A Src-selective tyrosine kinase inhibitor, 4-Amino-5-(4-methylphenyl)-7-(t-butyl) parasol (3,4-d)-pyrimidine (PP1) from Biomol was used at final concentration of 10 µM for 1 hr. A specific inhibitor of Rac GTPase, N6-[2-[[4-(Diethylamino)-1-methylbutyl]amino]-6-methyl-4-pyrimidinyl]-2-methyl-4,6-quinolinediamine trihydrochloride (NSC23766), from Tocris Bioscience (Ellisville, Missouri), was used at final saturating concentration of 100 µM for 1 hr. Platelet-derived growth factor (PDGF) (Sigma; St. Louis, MO) was used at final saturating concentration of 10 ng/ml. Methyl-β-cyclodextrin (MβCD), was used at a concentration of 10 mM to extract cholesterol from the plasma membrane. All cytoskeletal inhibitors were purchased from Sigma.

#### Rac Biosensor

**Figure 1:**
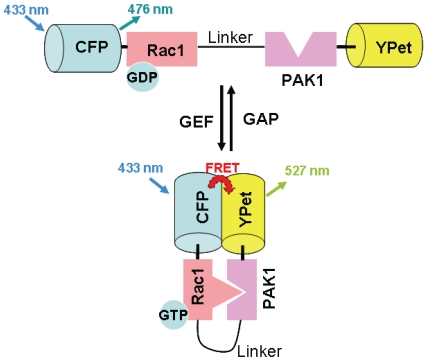
Quantification of Rac activity in a living cell by FRET. Top, the Rac GTPase (Rac1) and its substrate PAK1 are connected by a flexible linker. Rac1 is associated with Cyan fluorescent protein (CFP) and its substrate PAK1 (p21-activated kinase 1) (a serine-threonine kinase activated by Rac GTPase) with YPet (a variant of yellow fluorescent protein). When Rac is not activated, excitation at 433 nm yields an emission of 476 nm via CFP, no FRET occurs. Bottom, when Rac is activated by GEF (guanine nucleotide exchange factor) that stimulates GTPases by catalyzing the exchange of guanosine diphosphate (GDP) for guanosine triphosphate (GTP), Rac1 proceeds with conformational changes so that it binds specifically with its substrate PAK1. The binding of Rac1 with PAK1 leads to close association of CFP with YPet. Excitation of CFP at 433 nm now yields an emission of 527 nm via YPet, FRET occurs. In the presence of GAP (guanosine triphosphatase (GTPase)-activating protein) that hydrolyses GTP to GDP, Rac1 dissociates from PAK1, the Rac biosensor returns to inactivated and extended form. (Modified from [6]).

The Rac biosensor has been improved in its sensitivity by replacing the original fluorescence proteins with ECFP (as a donor for FRET) and YPet (as an acceptor for FRET) [4] (Fig. 1), based on a Rac biosensor pRaichu-Rac/RacCT, a gift from Dr. Michiyuki Matsuda at Kyoto University [6]. The probe of pRaichu-Rac/RacCT has been very well characterized in terms of its specificity [6]. The probes were transfected into HASM cells by adding 3 µg per 35-mm dish or SYF−/− MEF cells by adding 1 µg per 35-mm, both using Lipofectamine, following protocols provided by the manufacturer (Invitrogen) and incubated for 24 hours. A mutant Rac biosensor pc'-Rac(N17)-YPet was used as a negative control, where Thr^17^ of Rac1 was replaced by Asn, as previously described in [6].

#### Microscopy

A Leica inverted microscope was integrated with a magnetic twisting device and a Dual-View system (Optical Insights, Tucson, AZ) to simultaneously acquire both CFP and YFP emission images in response to stress. For emission ratio imaging, the Dual-View Micro-Imager (Optical Insights) was used. The CFP/YPet Dual EX/EM (FRET) (OI-04-SEX2) has the following filter sets: CFP: excitation, S430/25, emission S470/30; YPet: excitation, S500/20, emission S535/30. The emission filter set uses a 515 nm dichroic mirror to split the two emission images. Cells were illuminated with a 100W Hg lamp. For FRET imaging, each CFP (1344 pixels by 512 pixels) and each YPet image (1344 pixels by 512 pixels) were simultaneously captured on the same screen using a CCD camera (Hamamatsu C4742-95-12ERG) and a 40X 0.55NA air or a 63X 1.32NA oil-immersion objective.

#### Optical Magnetic Twisting Cytometry

Optical magnetic cell twisting is an extension of the magnetic cell twisting technique [7], [8] to any modes of forcing. The technique of applying twisting torques to cells in a dish under a microscope had been described in detail [9]. The microscope stage was heated to maintain 37°C for the cells in a dish. The twisting current was driven by a current source controlled by a computer. Ferromagnetic beads (∼4 µm diameter) coated with saturated amount of Arg-Gly-Asp (RGD)-containing peptides (the ligand density on the bead was measured to be about 1 RGD-peptide per 2 nm^2^ of bead surface area), ligands for integrin receptors, were bound to the surface of the adherent HASM cells for 15 min. Similar technique was used to coat the ferromagnetic beads with transferrin or poly-L-lysine, both at 50 µg/ml per mg bead. The binding specificity of bead binding was determined following protocols described previously [8]. The magnetic moments of each batch of self-made ferromagnetic beads were calibrated according to published methods [8]. The beads were magnetized by a strong (1,000 G) and short (<100 µs) magnetic field pulse oriented at the horizontal direction using the magnetizing coils. Then a step-function field (5, 10, 25, or 50 Gauss, equivalent of 1.8, 3.6, 8.8 or 17.5 Pa stress) was applied. A two-tailed Student's t-test was used for statistics.

#### Image Processing

A customized Matlab (Mathworks) program was used to obtain YPet/CFP emission ratios. CFP and YPet images at each time point were first background-subtracted and the YPet image was used to generate a binary mask based on an input threshold so that the pixel value inside the cell was set to 1 and the pixel value outside the cell was set to 0. After multiplication of the original YPet image by the mask image, this updated YPet image and the CFP image were aligned pixel-by-pixel by maximizing the normalized cross-correlation coefficient of the CFP and YPet images [10]. Aligned YPet/CFP emission ratios were normalized to the lower emission ratio and displayed as a linear pseudocolor. Since Rac activation occurs only at the cell periphery, the signal to noise ratio was increased by obtaining the normalized YPet/CFP emission ratio at 2 µm annulus around the cell boundary. A binary mask of the cell with the inside set to 1 was obtained by a separate customized Matlab program using the gray scale images with black background that was previously generated. The same image was then scaled down by 2 µm on all sides while maintaining the cell center of mass and image aspect ratio. A new binary mask was then obtained with the area outside of the cell set to 1. The original gray scale image was multiplied by the first and second binary masks yielding a 2-µm thick gray scale cell annulus image. The mean intensity of the gray scale cell annulus was then calculated as YPet/CFP emission ratio.

#### Experimental Protocols

After HASM or SYF−/− MEF cells were plated on collagen-1 coated dishes overnight, the cells were transfected with CFP-YPet Rac biosensors for 24 hrs. A single RGD-coated magnetic bead was attached to the cell apical surface for 15 min to allow for integrin clustering and formation of focal adhesions [8]. Then the bead was magnetized in the horizontal direction for a very brief period (<100 µs). A vertical, homogeneous, constant magnetic field was subsequently applied to the bead to generate rotational forces on the bead. The Rac activity was visualized simultaneously by collecting dual images of CFP and YPet of the cell on the same screen using the Dual View system. Alternatively, PDGF was added to the cell-containing medium and Rac activity was quantified.

## Results

**Figure 2:**
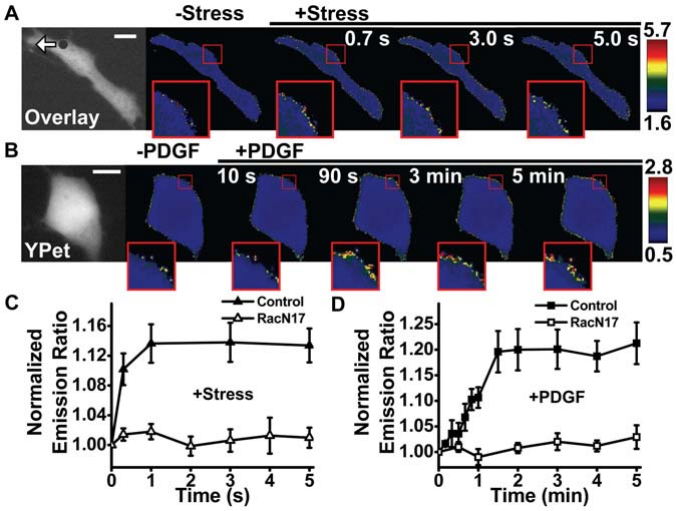
Rapid Rac activation in response to a local mechanical stress. (A) A 4.5-µm RGD-coated ferromagnetic bead was attached to the apical surface of the cell (black dot is the bead) for 15 min to allow integrin clustering and formation of focal adhesions around the bead. The bead was magnetized horizontally and subjected to a vertical magnetic field (step function) which applies a mechanical rotational stress (apparent average stress = 17.5 Pa) to the cell. A genetically encoded, CFP-YPet cytosolic Rac reporter was transfected into the smooth muscle cells following published procedures. The cytosolic Rac reporter was uniformly distributed in the cytoplasm (YPet fluorescence; white arrow indicates bead movement direction). The stress application induced rapid changes (<0.3 s) in FRET of the Rac reporter at discrete, distant sites at the cell periphery (the focal plane is ∼1 µm above cell base), indicating rapid Rac activation (see Insets). Images are scaled and regions of large FRET changes (strong Rac activity) are shown in red/yellow. Scale bar  = 10 µm. (B) Time-lapse images of Rac activation at the cell periphery after addition of PDGF (10 ng/ml) shows that activation of Rac in a representative cell by soluble factor PDGF is slow. Note that significant Rac activation occurred only at 0.5–1 min after PDGF treatment. Insets are enlarged areas showing Rac activation at the cell periphery. Scale bar  = 10 µm. (C) Normalized emission ratio of FRET as a function of stress application duration for the Rac biosensor (control) and its mutant form(RacN17) (Control, n = 6 cells; RacN17, n = 3 cells; mean +/− s.e.). A representative cell was shown in A with the Rac biosensor. (D) Normalized YPet/CFP emission ratio time courses of the Rac biosensor (Control) and the mutant Rac biosensor (RacN17) in response to PDGF treatment. (Control, n = 4 cells; RacN17, n = 3 cells; mean +/− s.e.).

We first determined how fast Rac could be activated by applied stress. As soon as the local mechanical stress (17.5 Pa, step function) was applied, the Rac GTPase was activated within 300 ms, as evidenced by the significant changes in FRET at the cell peripheries (Fig. 2A, C). It is interesting that the Rac activation occurs only at some sites on the cell surface (see insets in Fig. 2A). In contrast, the PDGF (10 ng/ml) induced Rac activation only occurred ∼30 sec after the growth factor treatment (Fig. 2B, D). This suggests that force-induced Rac activation is much faster than growth factor induced signal transduction, consistent with the published results in Src activation [11]. To determine the specificity of the Rac biosensor, we transfected a mutant form of the biosensor RacN17 into the cells [7]. Neither stress nor PDGF activated the RacN17 biosensor, suggesting that the activation of Rac by stress or PDGF was specific to the Rac molecule.

**Figure 3:**
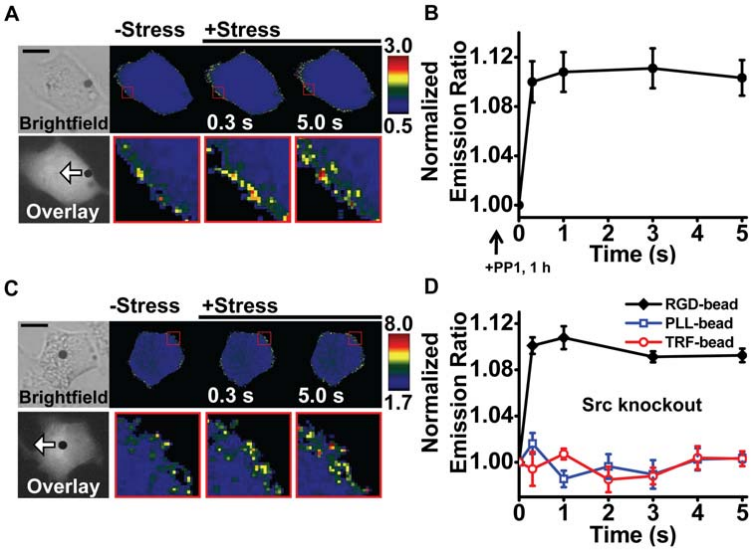
Stress-induced Rac activation is independent of Src activity. (A) Time-lapse images of a representative smooth muscle cell transfected with the Rac biosensor and pretreated with a specific Src inhibitor, PP1 (10 µM for 1 hr), before stress application. Florescence resonance energy transfer (FRET) changes were observed within 0.3 s after stress application as shown in the inset with enlarged cell periphery. White arrow indicates magnetic bead movement direction when a stress of 17.5 Pa is applied. Scale bar  = 10 µm. (B) Average data showing rapid Rac activation by stress even though Src was inhibited by PP1. (n = 6 cells, mean +/− s.e.). (C) Time-lapse emission ratio images of the Rac biosensor in response to 17.5 Pa stress in a representative Src/Yes/Fyn triple-knockout (SYF−/−) MEF cell. The inset with the enlarged area of the cell periphery shows rapid activation of Rac. White arrow indicates RGD coated magnetic bead movement direction. Scale bar  = 10 µm. (D) Probe specificity of Rac activation. Normalized YPet/CFP emission ratio shows that Rac activation is induced in SYF−/− mouse MEFs by mechanical stress applied via RGD-coated magnetic beads that bind specifically to integrin receptors (n = 6 cells). Although the PDGF-induced Rac activation is known to be downstream of Src [4], knocking-out Src did not prevent Rac activation by stress. However, application of the same magnitude of stress at 17.5 Pa with beads coated with transferrin (TRF-bead) or poly-L-lysine (PLL-bead) did not activate Rac (TRF-bead, n = 4 cells; PLL-bead, n = 4 cells). Mean +/− s.e.

Since it has recently been shown that the PDGF-induced Rac activation is dependent on Src activation [4], we wondered whether the stress-induced Rac activation was different or not. To determine this, we pretreated the cell with a specific Src inhibitor, PP1 (10 µM for 1 hr) before stress application. Surprisingly, there was no change in stress-induced Rac activation despite the fact that Src was inhibited (Fig. 3A, B, comparing to Fig. 2A, C). This result is drastically different from the PDGF-induced Rac activation which is blocked by Src inhibition with PP1 [4]. To further confirm our results from PP1-treated cells, we measured Rac activation in Src/Yes/Fyn triple-knockout (SYF−/−) mouse embryonic fibroblasts (MEFs). The same stress induced similar Rac activation at the cell periphery (Fig. 3C, D). These results are fundamentally different from the recent finding that the PDGF-induced Rac activation was inhibited in these SYF−/− MEFs [4]. The stress-induced Rac activation was specific for the integrin-cytoskeleton structural pathway, since the same magnitude of stress applied with magnetic beads coated with transferrin or poly-L-lysine failed to activate Rac (Fig. 3D).

**Figure 4:**
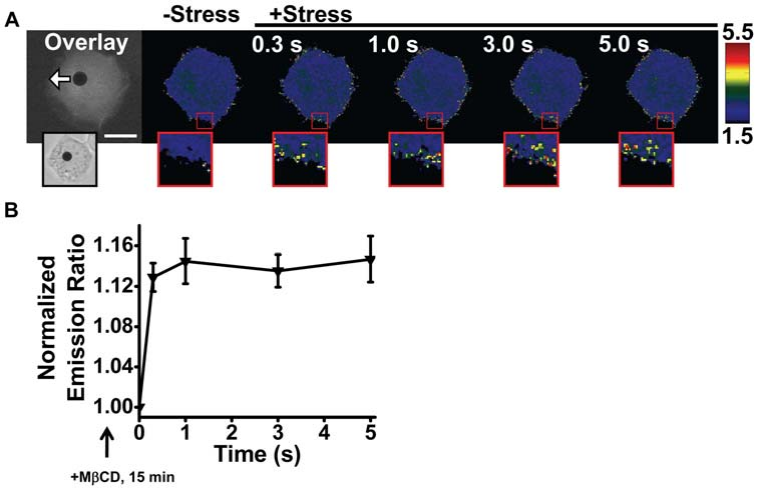
Rapid Rac activation despite extraction of cholesterol from the plasma membrane. (A) Representative time-lapse emission ratio images of a SYF−/− MEF cell treated with 10 mM of methyl-β-cyclodextrin (MβCD) for 15 min to selectively extract cholesterol from the plasma membrane before the application of stress. Inset shows the enlarged area of the cell periphery where rapid activation of Rac is observed. The left inset panel shows the brightfield image of the cell with a magnetic bead (black dot) bound to the apical surface. (B) Normalized emission ratio time course of SYF −/− MEFs in response to 17.5 Pa stress pretreated with methyl-β-cyclodextrin (MβCD). This shows that the activation of Rac occurs independent of the integrity of lipid rafts at the plasma membrane. (n = 4 cells, mean +/− s.e.).

Since Rac localizes at the cell membrane [12], we wondered if its activation depends on lipid rafts in the plasma membrane. We extracted cholesterol with methyl-beta-cyclodextrin (MβCD) (10 mM for 15 min) [13]. It is interesting that the pretreatment with MβCD did not abolish the stress-activated Rac (Fig. 4). This result indicates that the stress-induced Rac activation is independent of lipid rafts at the plasma membrane.

**Figure 5:**
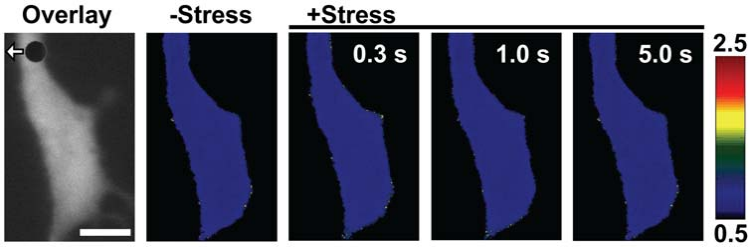
Rac activation by stress is specific. The cell was pretreated with a specific Rac inhibitor, NSC23766 (25 µM for 1 hr) before stress application. The rest of the protocol was the same as in Fig. 2 (A). There were no changes in FRET ratios in response to stress, suggesting that the drug prevented stress-induced Rac activation. Two other cells showed similar behavior. Left panel shows the YPet fluorescent image of the cell with the magnetic bead overlaid on top. White arrow represents bead movement direction. Scale bar = 10 µm.

To further test the specificity of the stress-induced Rac activation, we pretreated the cell with a known specific Rac inhibitor, NSC23766 (100 µM for 1 hr) [14]. Pretreatment with NSC23766 completely prevented the stress-induced Rac activation (Fig. 5). Together with the data with the mutant RacN17 biosensor in Fig. 2C, these results demonstrate that stress-induced Rac activation was not due to the unspecific conformational changes in the biosensor.

**Figure 6:**
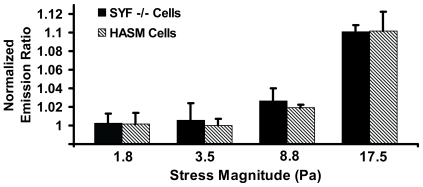
Rac activation is dependent on the stress-magnitude. Bar graphs represent the normalized emission ratios of the YPet/CFP Rac biosensor in human airway smooth muscle (HASM) cells and SYF−/− MEFs at the time point of 0.3 s upon stress application. There were no significant differences in Rac activation between 1.8 and 3.5 Pa stress, or between 3.5 and 8.8 Pa stress (p>0.05), but there were significant differences between 8.8 and 17.5 Pa stress (p<0.01), for either HASM or SYF−/− MEF cells. These data suggest that stress threshold for Rac activation is between 8.8 and 17.5 Pa. At 1.8 Pa, n = 6 or 7; at 3.5 Pa, n = 5 or 7; at 8.8 Pa, n = 5 or 8; at 17.5 Pa, n = 6; for HASM or SYF−/− MEFs, respectively. Mean +/− s.e.

If the Rac activation were directly induced by stress, it must depend on the magnitude of the applied stress. Indeed, our data showed that the stress threshold for Rac activation was between 8.8 and 17.5 Pa (Fig. 6). These results support the idea that Rac was rapidly activated by the stress applied via the magnetic bead bound to the integrin receptors.

## Discussion

The mechanism of mechanotransduction, i.e., how mechanical forces are converted into biochemical activities inside the cell is a central question in cell biology. Our findings demonstrate that the plasma membrane bound Rac at remote sites or even at opposite ends of the local stress application site (i.e., the site of the bead) can be activated directly and rapidly. This finding clearly challenges the established dogma in the field of cell biology. Our results also challenge the established classical continuum mechanics theory-St. Venant's principle that a local force can only cause a local deformation. Our results are consistent with a recently published report that Src protein is directly activated by local mechanical stresses [11] and action at a distance in living cells [15], [16]. However, there are differences between our study and the study done by Na *et al*. Src is a protein that localizes on the endosomal membranes, which in turn are connected with microtubules. Thus Src is directly activated via microtubule deformation [11]. In contrast, Rac is a plasma membrane bound protein. It is well known that the plasma membrane is rather fluid and thus dissipate forces very rapidly. Therefore, it is not clear how the plasma membrane-bound Rac is activated by a local force. An additional piece of evidence for the difference between Src and Rac is that the threshold stress for Rac activation is ∼5-fold higher than for Src activation (∼10 Pa for Rac, 1.8 Pa for Src) in the same human airway smooth muscle cell (Fig. 6; [ref. 11]). It is interesting that two different types of cells (fibroblasts and smooth muscle cells) exhibit similar threshold stresses for Rac activation, suggesting that the setpoint for the enzyme activation is the threshold strain (deformation), since both cell types have similar softness (or its inverse, stiffness). This interpretation is consistent with our recent findings in embryonic stem cells and differentiated cells that cell softness dictates biological responses of a living cell to a local stress [17].

We wondered if Rac activation can be triggered by diffusion or translocation of molecules. It is known that calcium is one of the fastest diffusive molecules in the cytoplasm with a diffusion coefficient of ∼60 µm^2^/s [18]. If calcium ion channels were opened as a result of the local mechanical force, then for a distance of 30 µm in the cytoplasm, it would take ∼4 s [t = square of distance divided by four times diffusion coefficient = (30 µm)^2^/[4×60 µm^2^/s] = 3.75 s] for calcium to reach Rac at the remote site. However, we observed that Rac activation occurs in less than 300 ms after force application. Therefore the activation speed is too rapid to be explained by calcium diffusion. Another possibility is protein translocation along the cytoskeleton to activate Rac. It is known that translocation speed is <4 µm/s in the cytoplasm [19]. Hence it would take at least 7 s for the protein to be translocated across a distance of 30 µm. Again, this is too slow to explain the rapid activation of Rac that we have observed in living cells.

**Figure 7:**
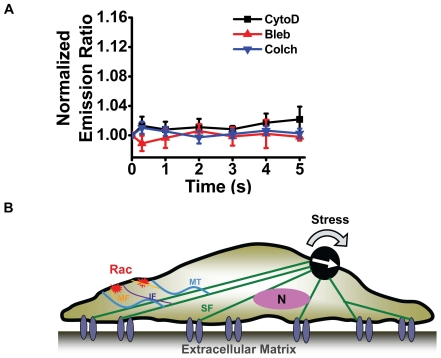
Cytosketal integrity is necessary for Rac activation by stress. (A) Inhibition cytoskeletal tension with blebbistatin (Blebb, 50 µM for 30 min, n = 7 cells), disrupting F-actin with cytochalasin D (CytoD, 1 µg/ml for 15 min, n = 6 cells) or microtubules with colchicine (Colch, 10 µM for 15 min, n = 9 cells), blocks stress-induced Rac activation in SYF−/− MEFs. Mean+/−s.e. (B) A working model for rapid Rac activation by stress. A local load (magnetic bead) applied to focal adhesions leads to stress propagation along the actin bundles (red lines) without decay in magnitudes at remote sites. Rac GTPase bound to the plasma membrane at the other end of the cell are activated rapidly when stress waves reach the plasma membrane via the cytoskeleton to directly deform Rac, causing a conformational change in the enzyme. MF =  actin microfilament; MT = microtubule; IF = intermediate filament; SF = stress fiber; N = nucleus (not drawn to scale). Black dot = the magnetic bead. White arrow = magnetic moment direction of the magnetized bead. Curved black arrow = the rotational shear stress.

Alternatively, it is possible that a local force applied to integrin receptors propagates along the tensed actin bundles and reach remote sites in the cytoplasm within 1 ms through elastic wave propagation without significant decreasing in stress magnitudes [11], which in turn leads to a deformation of cytoskeleton filaments such as microfilaments, microtubules and intermediate filaments [20]. This model of the integrin-cytoskeleton force transduction pathway is supported by the data that inhibiting myosin-II dependent tension with blebbistatin, disrupting F-actin with cytochalasin D, or disrupting microtubules with colchicine, all blocked stress-induced Rac activation (Fig. 7A). Some of these cytoskeletal networks may form strong connections with the plasma membrane at certain sites. It is at these sites that Rac GTPase is activated. A working model for force-induced rapid Rac activation is illustrated in Fig. 7B.

It is known that uniaxially stretching a whole cell by a flexible substrate increases Rac activation, possibly by tension, whereas at the lateral sites Rac activity is inhibited, possibly by compression [21]. Since we apply a rotational shear stress to the cell surface via the magnetic bead, it is possible that the Rac activation sites in our cells are the sites where local tension is increased. This possibility needs to be tested in future studies.

Future work is also needed to determine the role of substrate stiffness in regulating the stress-induced Rac activation [22]. Furthermore, to test our working model for the stress-induced direct Rac activation, the structural basis for Rac activation needs to be elucidated to find out which cytoskeletal filament systems are the most important in mediating the propagation of stresses to activate Rac. In addition to Src, it is shown that phosphatidylinositol–3-kinase (PIP3 kinase) can facilitate recruitment of Rac to cell membrane [23]. Since Rac and Rho proteins are negative regulators of each other [24], it remains to be seen if the stress-induced Rac activation leads to inhibition of Rho at the cell periphery.

In summary, we have found that a local stress of physiologic magnitude can directly activate Rac GTPase rapidly, independent of the Src activity. In principle our approach of combining FRET with a local stress application can be extended to any molecules in a living cell [25]. Our finding on the stress-induced rapid Rac activation challenges the conventional wisdom on mechanotransduction and suggests that stress-induced signaling does not follow signal transduction pathways induced by growth factors.
